# Interleukin-17 promotes angiogenesis by stimulating VEGF production of cancer cells via the STAT3/GIV signaling pathway in non-small-cell lung cancer

**DOI:** 10.1038/srep16053

**Published:** 2015-11-03

**Authors:** Bo Pan, Jing Shen, Jingyan Cao, Yongxu Zhou, Lihua Shang, Shi Jin, Shoubo Cao, Dehai Che, Fang Liu, Yan Yu

**Affiliations:** 1 Department of Medical Oncology, Harbin Medical University Cancer Hospital, Harbin 150081, PR China; 2 Department of general surgery, The Fourth Affiliated Hospital of Harbin Medical University, Harbin 150001, PR China

## Abstract

The presence of IL-17-positive cells is observed in a variety of inflammatory associated cancers and IL-17 has been found to be involved in angiogenesis. However, it remains unclear how IL-17 might contribute to tumor angiogenesis. In our study, IL-17 enhanced the formation of vessel-like tubes in HUVECs both directly (when HUVECs were incubated with IL-17) and indirectly (when HUVECs were incubated in conditioned cell media (CCM) from IL-17-treated cancer cells). Our results from experiments using siRNA-mediated knockdowns of STAT3 and GIV suggest that the effects of IL-17 were mediated by activating STAT3/GIV signaling in NSCLC cells and subsequently up-regulating its downstream target VEGF. Consistent with these findings, immunostaining experiments on human NSCLC tissues indicated that IL-17 and GIV expression were significantly and positively associated with increased tumor vascularity. The clinical significance of IL-17 was authenticated by our finding that the combination of intratumoral IL-17 + cells and GIV expression served as a better prognosticator for survival than either marker alone. Therefore, our finding highlights a novel aspect of STAT3/GIV pathway in the IL-17 promotes tumor angiogenesis of NSCLC.

Non-small-cell lung cancer (NSCLC) accounts for 80–85% of total lung malignancies^1^.The outcome of NSCLC is poor and the disease is rarely curable. The overall five-year survival rate is less than 15%^2^ and is largely due to lung cancer cell metastasis^3^,^4^. Angiogenesis is a critical hallmark of malignancy and can occur at different stages of the tumor progression^5^. Angiogenesis is regulated by a balance between pro- and anti- angiogenesis factors, and the disruption of this balance contributes to the pathogenesis of numerous disorders including cancer^6^.

T helper 17 (Th17) cells are an important inflammatory component whose main physiological role is to promote host defense against infectious agents. Th17 cells are well known for their role in contributing to autoimmune diseases^7^. Recently, Th17 cells and their signature cytokine, interleukin-17 (IL-17), have been found to be present in increased frequencies within certain tumors^8^,^9^,^10^. Chang and colleagues has demonstrated a critical role for Th17 cell-mediated inflammation in lung tumorigenesis^11^. In our previous study, we found that serum IL-17 was elevated and the levels positively correlated with VEGF concentration in NSCLC patients^12^. Consistently, transfection of IL-17 into tumor cells augmented the progression of the disease in nude mice via effects on the vascular endothelium and increased neoangiogenesis^13^,^14^. However, IL-17’s mechanisms underlying its modulation of human NSCLC cell angiogenesis remain elusive.

Accumulating evidence is defining Signal transducer and activator of transcription 3 (STAT3) as an important pathway for signal transduction in cancer metastasis and angiogenesis^15^,^16^. GIV(Gα-Interacting Vesicle-associated protein, also known as Girdin) is a guanidine exchange factor (GEF) that modulates key signaling pathways during a diverse set of biological processes such as wound healing, macrophage chemotaxis, cancer invasion/metastasis and tumor angiogenesis. GIV is a direct target of the STAT3 in breast cancer cells^17^. Others have reported that GIV is expressed exclusively in colorectal carcinoma cells with high metastatic potential and is virtually undetectable in those with poor metastatic potential, implying the involvement of GIV in tumor metastasis^18^. Here, we speculate that GIV may play a role in the angiogenesis of cancer cells. In this study, we attempted to elucidate the exact role and associated molecular mechanism of IL-17 in NSCLC angiogenesis. The clinical relevance and prognostic significance of IL-17 in human NSCLC were also investigated.

## Results

### IL-17 is positively correlated with MVD in human NSCLC tissues and enhanced formation of vessel-like tubes in HUVECs

**Figure 1:**
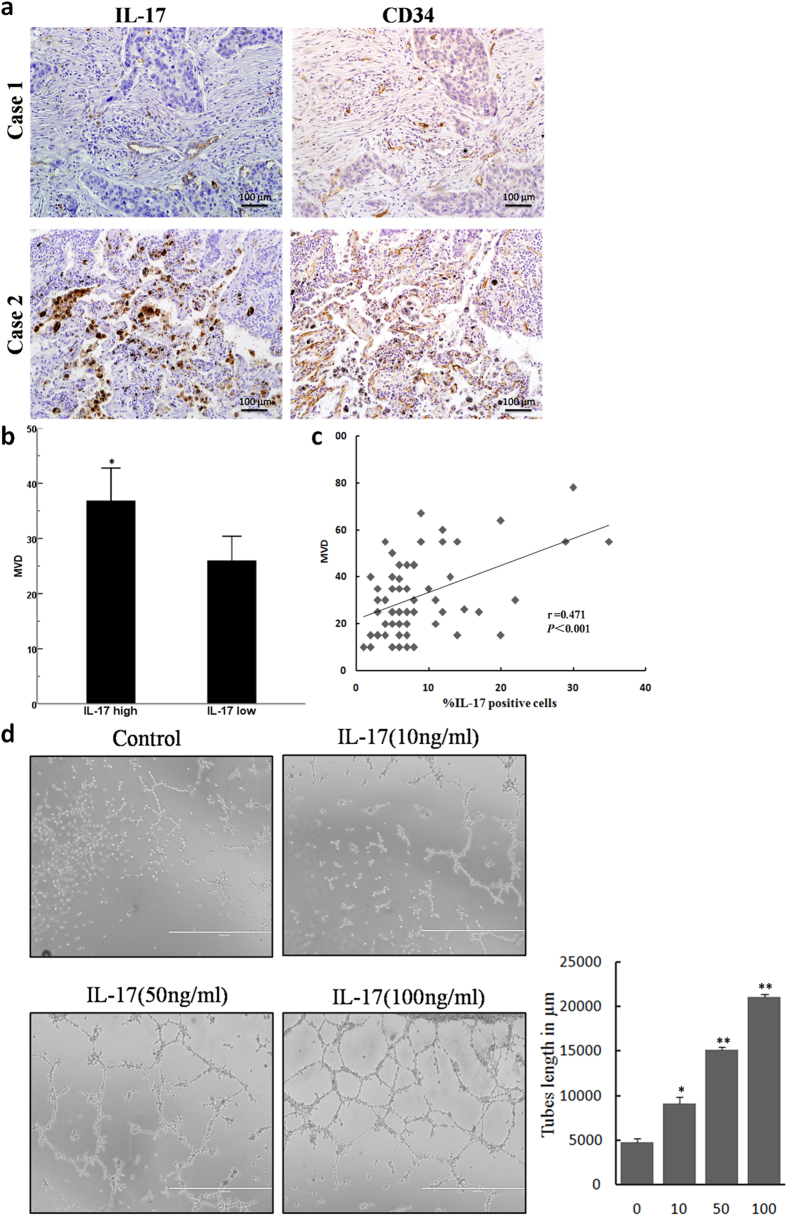
IL-17 expression is associated with MVD, and IL-17 promotes *in vitro* tube formation in HUVECs. (**a**) IL-17-positive cells expression and MVD staining for CD34 in NSCLC tissues (magnification, 200×). (**b**) Quantification of stains of immunohistochemistry; 5 random high-powered fields per section were counted for number of CD34-stained vessels intensity and distribution; Date are expressed as means; Student′s *t* test; ^*^p < 0.05. (**c**) Significant positive correlations were found between the IL-17 expression and MVD. Spearman′s rank correlation coefficient; r = 0.471; *p* < 0.001. (**d**) Representative photographs (left panel) and mean numbers of tube length (right panel) at ×100 magnification. HUVECs were seeded on Matrigel-coated plates incubated with IL-17 (10 ng/ml, 50 ng/ml or 100 ng/ml) or vehicle control at 37 °C for 8 h (n = 3). *p < 0.05.

High densities of h17 cells infiltrating tumours have been associated with increased angiogenesis in studies from human gastric^19^, colorectal^20^, hepatocellular^21^, and pancreatic cancers^22^. In addition, the level of IL-17-producing cells has been positively correlated with MVD in a tumor-bearing mouse model^23^. To investigate the role of IL-17 in angiogenesis in patients with NSCLC, we stained consecutive sections in 67 NSCLC patients (Fig. 1a). We found that the majority of IL-17 staining was localized to the cytoplasm of mononuclear cells in NSCLC tissues. Our results indicated that patients with high IL-17 expression exhibited high MVD (*p* = 0.016, Fig. 1b). Moreover, a correlation analysis revealed a significant positive correlation between the density of IL-17-producing cells and MVD (r = 0.471; *p* < 0.001, Fig. 1c).

Next, we examined this cytokine’s effect on tube formation by HUVECs. HUVECs were treated with recombinant human IL-17(rhIL-17) or a vehicle control. As expected, treatment with IL-17 promoted the formation of vessel-like tubes in a dose-dependent manner (Fig. 1d). Quantitative analysis of endothelial cell networks revealed that IL-17 significantly increased the tube length compared to control cultures. These findings demonstrated that IL-17 plays a potential role in promoting angiogenesis.

### IL-17 activates STAT3 in NSCLC cells

**Figure 2:**
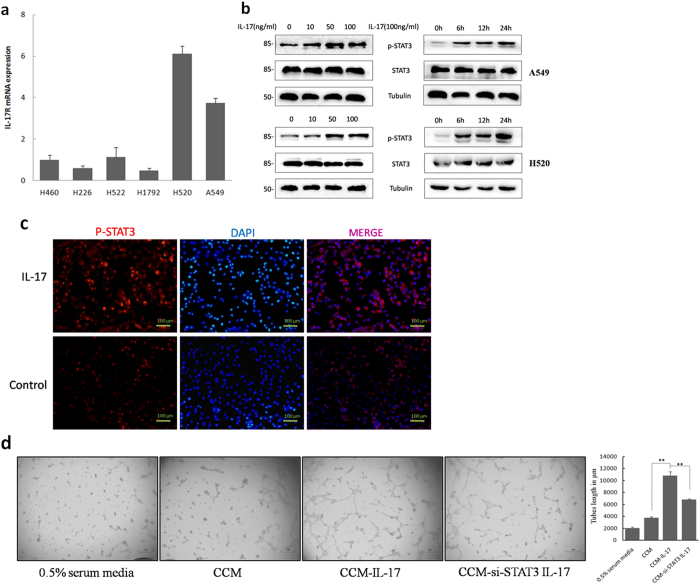
IL-17 promotes NSCLC angiogenesis via STAT3 activation. (**a**) mRNA expression of IL-17R in NSCLC cell lines. (**b**) Western blotting showed that phosphorylation of STAT3 were obviously increased as early as 6 h after IL-17 treatment and lasted for 24 h after IL-17 stimulation. A549 cells were incubated with IL-17 at the indicated concentrations for 24 h or at 100 ng/ml for the indicated time. (**c**) Immunofluorescence assays showed that recombinant human IL-17(100 ng/ml for 24 h) significantly elevated the expression of p-STAT3 in A549 cells. Photomicrographs were taken at ×200 magnification. Control, PBS. (**d**) A549 cells or A549-siRNA-STAT3 cells were treated with IL-17 at 100 ng/ml for 24 h, media was harvested, added to HUVECs plated on Matrigel. HUVEC were seeded in 96-well plates coated with matrigel and treated with CCM or CCM-IL-17 from A549 cells or A549-siRNA-STAT3 cells for 16 h. In this experiment, HUVEC incubated with 0.5% serum containing 1640 media served as a negative control. Tubular structures were photographed at 40× magnification and tube length was measured as described in ‘Materials and Methods’. Tube length data is presented as mean ± standard deviation of three samples for each treatment.

IL-17 is known for its effects on angiogenesis being reliant on the surrounding endothelial cells and fibroblasts. In our present study, we found expression of the IL-17 receptor (IL-17R) at the mRNA level in all detected NSCLC cell lines (Fig. 2a). Because STAT3 activation in tumor cells plays a critical role in tumor progression by augmenting tumor survival and tumor angiogenesis and by suppressing antitumor immunity^24^, we explored the possibility mechanism that IL-17 mediates tumor angiogenesis via activation of STAT3 in tumor cells. Here, we cultured A549 cells and H520 cells with or without recombinant human IL-17 and found that IL-17 stimulated increased phosphorylation of STAT3 *in vitro* as early as 6 h after IL-17 treatment. This effect lasted for 24 h (Fig. 2b). Furthermore, this increased phosphorylation was confirmed by immunofluorescence assays tumor cells that were cultured for 24 h in the presence or absence of IL-17. IL-17 treatment of NSCLC cells markedly increased p-STAT3 expression (Fig. 2c and Fig. S1).

To explore the potential role of STAT3 in IL-17-mediated effects on NSCLC angiogenesis, STAT3 expression was reduced by small interfering RNA (siRNA) (Fig. S2). A549 and H520 tumor cells were exposed to STAT3 siRNA (A549-siRNA-STAT3 or H520-siRNA-STAT3) or empty vector and then treated with rhIL-17 at 100 ng/ml for 24 h. Conditioned cell media (CCM) from these cells were harvested and then added to HUVECs plated on Matrigel. We observed that CCM from rhIL-17-treated A549 cells strongly enhanced tube formation by HUVECs. On the other hand, in HUVECs plated with CCM from rhIL-17-treated A549-siRNA-STAT3 cells, the ability of IL-17 to promote tube formation was diminished, as evidenced by decreased tube length (Fig. 2d). These results suggest an important contribution from STAT3. We observed similar results regarding tube formation in experiments with CCM collected from H520 cells and H520-siRNA-STAT3 cells.

### IL-17 promotes NSCLC angiogenesis via the STAT3/GIV signaling pathway

**Figure 3:**
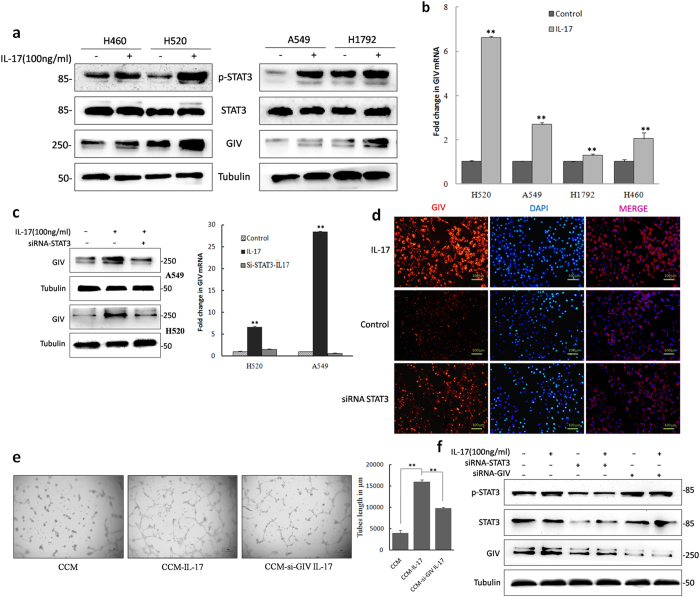
IL-17 induces the expression of GIV and GIV participated IL-17 mediated angiogenesis in NSCLC cells. (**a**) A549, H520, H1792 and H460 cells were treated with IL-17(100 ng/ml for 24 h) and expression levels of GIV, p-STAT3, and STAT3 were examined by Western blotting. (**b**) mRNA level of GIV were examined in tumor cells treated with IL-17 by real-time PCR. (**c**) A549 and H520 cells were cultured for 24 h with IL-17 or PBS. The protein and mRNA levels were measured by western blotting and qPCR. Depletion of STAT3 markedly reversed IL-17-mediated GIV expression increased both protein and mRNA levels. (**d**) Immunofluorescence assays showed that recombinant human IL-17(100 ng/ml for 24 h) significantly elevated the expression of GIV in A549 cells. However, the expression of GIV decreased if depleted of endogenous STAT3. The photomicrographs were taken at ×200 magnification. **(e)** HUVECs were seeded in 96-well plates coated with matrigel and treated with CCM or CCM-IL-17 from A549 cells or A549-siRNA-GIV cells for 16 h. Tubular structures were photographed at 40× magnification. Tube length data is presented as mean ± standard deviation of three samples for each treatment. (**f**) H520 or H520-siRNA-STAT3 or H520-siRNA-GIV cells were incubated with or without IL-17, and Western blotting were performed for phosphor-STAT3, total STAT3, GIV and tubulin protein levels.

GIV is widely expressed in a variety of cancer cell lines, including MDA-MB-231 (estrogen-independent breast carcinoma cell line), Ls-174T (colon cancer cell line), A431 (skin squamous cell carcinoma cell line), HeLa (uterine cervical carcinoma cell line), and HT-1080 (fibrosarcoma cell line)^25^,^26^. Some experiments have shown that GIV is positively correlated with increasing tumor aggressiveness and angiogenesis^27^. In our study, western blot analysis of GIV expression in NSCLC cell lines was used to define the role of GIV in NSCLC progression (Fig. S3). IL-17 treatment of tumor cells markedly increased p-STAT3 expression (Fig. 3a) as well as GIV expression (Fig. 3a,b). Ying Dunkel *et al.* demonstrated that GIV expression is upregulated due to increased transcription mediated directly by STAT3^17^. Consistent with this result, IL-17 significantly elevated the expression of GIV in A549 and H520 cells. However, depletion of STAT3 markedly reversed IL-17-mediated GIV expression changes at both the protein and mRNA levels in A549 and H520 cells (Fig. 3c). Furthermore, immunofluorescence assays showed that IL-17 treatment of cells clearly upregulated the expression of GIV (Fig. 3d and Fig. S4). Conversely, depletion of endogenous STAT3 resulted in decreased GIV expression. To further investigate the role of GIV on IL-17-mediated tumor angiogenesis, we depleted GIV in A549 and H520 cells (Fig. S5). In HUVEC tube formation assays, CCM from A549 cells treated with IL-17 significantly enhanced tube formation. GIV-siRNA significantly reversed IL-17-stimulated tube formation in A549 cells (Fig. 3e). These findings demonstrate that STAT3 positively regulates GIV expression and that IL-17 promotes NSCLC angiogenesis via STAT3/GIV activation.

Others have previously demonstrated that GIV positively autoregulates its own transcription by enhancing STAT3 activation via its GEF motif in invasive cancer cells^17^. In our study, STAT3 increased GIV expression; depleted STAT3 or GIV in NSCLC cells diminished IL-17-mediated angiogenesis in a microenvironment. However, we did not find that GIV enhances STAT3 activation in NSCLC cells (Fig. 3f).

### *IL-17 induces* VEGF production via STAT3/GIV activation in NSCLC cells

**Figure 4:**
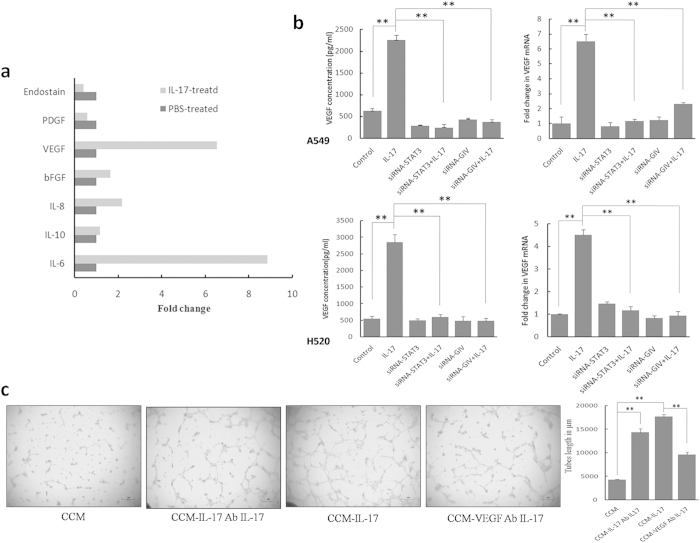
IL-17 stimulates NSCLC cells to produce VEGF via STAT/GIV activation. (**a**) A549 cells were treated with PBS or with IL-17 for 24 h. The mRNA levels of proangiogenic factors were analyzed with Realtime-PCR. (**b**) NSCLC cells were cultured for 24 h with IL-17(100 ng/ml) or PBS. Concentrations of VEGF in culture supernatants were measured by ELISA, and mRNA of VEGF in NSCLC cells were measured by Real-time PCR. IL-17 up-regulated the production of VEGF by tumor cells. Both siRNA-STAT3 and siRNA-GIV significantly downregulated the expression of VEGF. **(c)** A549 cells were cultured for 24 h with IL-17(100 ng/ml) in the presence or absence of VEGF mAb (20 ng/ml), then the CCM harvested. Before CCM added to HUVECs, IL-17 mAb(500 ng/ml) was added to the IL-17 treatment medium. Tubular structures were photographed at 40× magnification. Tube length data is presented as mean ± standard deviation of three samples for each treatment.

IL-17 is known for eliciting secretion of diverse inflammatory mediators in diverse cell types, including stromal cells and tumor cells. We hypothesized that IL-17 might induce the expression of proangiogenic factors(s) by cancer cells that then act on endothelial cells to promote angiogenesis. We found that IL-17 selectively up-regulates IL-6 and VEGF protein expression in all NSCLC cell lines studied (A549, H520, H1792, H460 cells; Table 1 and Table S1). We observed a similar increase in mRNA levels in A549 cells. The most prominent increase in secretion was for IL-6, with an 8.8-fold increase, followed by VEGF (6.51-fold) in A549 cells (Fig. 4a). By contrast, the production of IL10, IL-8, bFGF, PDGF and Endostain were not significantly affected by exogenous IL-17.

**Figure 5:**
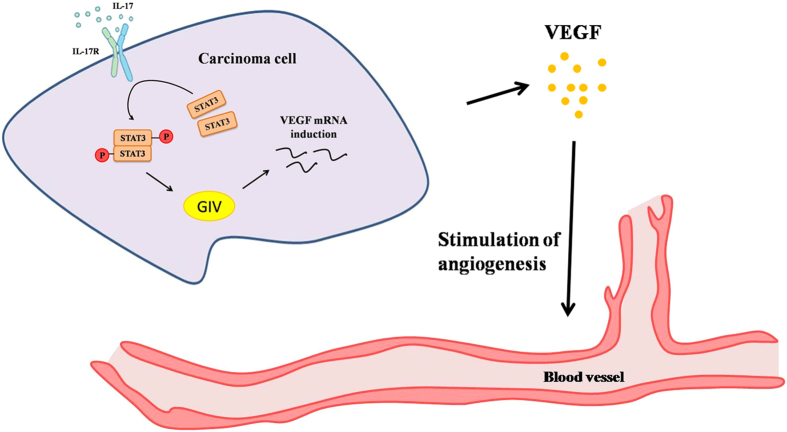
IL-17-mediated tumor angiogenesis involves activation of the STAT3/GIV signaling pathway and subsequent up-regulation of VEGF production in NSCLC cells.

VEGF is one of the most important angiogenic factors. We postulated that IL-17 might affect tumor cells’ production of VEGF by activating the STAT3/GIV pathway in tumor cells. Notably, in A549-siRNA-STAT3 cells and H520-siRNA-STAT3 cells, IL-17-induced expression of VEGF was significantly inhibited (Fig. 4b). Meanwhile, GIV-siRNA significantly reversed IL-17-induced VEGF expression at both the protein and mRNA levels in NSCLCs (Fig. 4b), while Il-17-induced IL-6 up-regulation was not affected (Fig. S6). Furthermore, HUVEC cells plated with CCM from A549 cells, neutralizing VEGF by using blocking mAb significantly reduced IL-17-induced tube formation, while using IL-17 mAb treated were not affected (Fig. 4c). These results indicated that VEGF is an important target gene in the STAT3/GIV pathway. Thus, VEGF may be the main downstream target of STAT3/GIV in the context of IL-17-induced tumor angiogenesis (Fig. 5).

IL-17 has recently been reported to activate STAT3 via IL-6 induction^28^,^29^. Therefore, we determined whether IL-6 mediated IL-17-driven STAT3 activation in our study. After neutralizing IL-6 with a blocking mAb, IL-17 stimulation still significantly increased p-STAT3 expression in tumor cells even though STAT3 activation was diminished. The results of the tube formation assay also indicated a similar phenomenon in regards to treatment with IL-6 mAb (Fig. S7). Thus, our study showed that IL-6 may be a main upstream activator of pSTAT3 expression, but is not indispensable in the setting of IL-17 stimulation.

### GIV expression positively correlates with IL-17+ cell and microvessel densities and predicts poor survival of NSCLC patients

**Figure 6:**
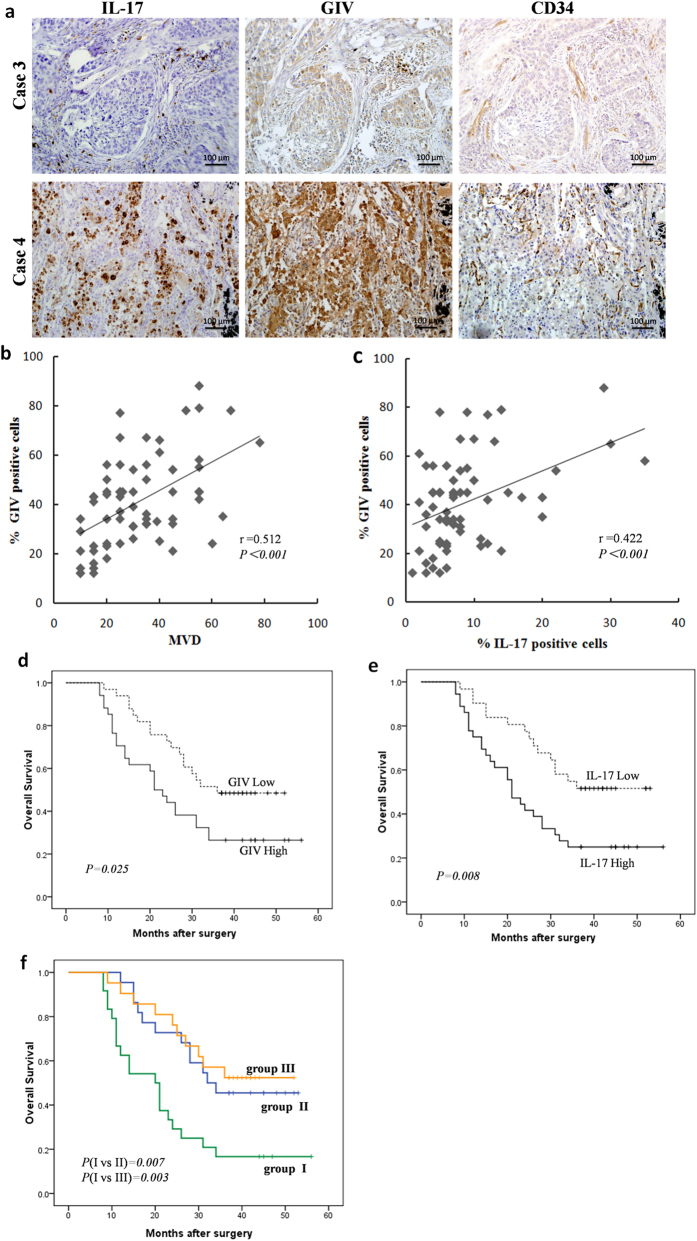
IL-17 and GIV clinical relevance and is associated with poor prognosis. (**a**) Serial whole tumor sections from 67 NSCLC patients were used and representative cases of immunostaining of IL-17, GIV and CD34 (MVD) are shown (magnification, 200×). (**b**,**c**) Significant positive correlations were found between the GIV expression and MVD (**b**), GIV expression and IL-17-positive cells intensity (**c**). (**d**,**e**) Kaplan-Meier curves of overall survival analysis between NSCLC patients. GIV (**d**) or IL-17(**e**) with high expression patients had significantly poorer OS than low expression patients. (**f**) NSCLC patients were classified into three groups: group I, high expression both of IL-17+ cells and GIV; group II, high expression of one of the two markers; and group III, low expression of both markers. The 3 year survival rates in group I were significantly lower than those in groups II and III. P values were determined by log-rank test.

Because high densities of IL-17+ cell tumors have been associated with increased angiogenesis in our studies (Fig. 1b), we further explored whether a similar phenomenon exists regarding GIV in human NSCLC tissue. A correlation analysis on immunostained consecutive tissue sections from 67 NSCLC patients (Table S2) revealed significant positive correlations between GIV expression and MVD (r = 0.512; p < 0.001; Fig. 6a,b), as well as between GIV expression and densities of intratumoral IL-17-producing cells (r = 0.422; p < 0.001; Fig. 6c). Thus, GIV may play an important role in IL-17-induced tumor angiogenesis.

We next investigated the prognostic value of IL-17 and its target GIV in NSCLC patients. At the time of data analysis, 42 of the 67 patients had died of the disease during the follow-up period of 36 months. The median cumulative overall survival (OS) was 28 months, and the three-year OS rate was 37.3%. Patients with high expression of GIV had significantly poorer OS than those with low expression of GIV (Fig. 6d). Similarly, there was a significant inverse correlation between intratumoral IL-17+ cell density and patient survival (Fig. 6e). These results suggest that increased intratumoral IL-17+ cells and increased GIV expression are associated with NSCLC progression.

Univariate analysis revealed that intratumoral IL-17+ cells and GIV expression were significantly associated with survival (Table 2). Patients were classified into three groups: group I, high expression of both markers; group II, high expression of either marker; group III, low expression of both markers. Significant differences in survival were detected among the three groups (Fig. 6f). Subsequently, in a multivariate COX regression analysis where the presence of IL-17-positive cells, GIV expression, and the combination of both markers were simultaneously adopted as covariates (Table 2), only the combination remained statistically significant, whether the comparison was between group I and II or between group I and III. Furthermore, multivariate analyses showed that GIV expression independently correlated with, irrespective of being used alone or in combination (Table S3). These results collectively suggest that intratumoral IL-17+ cells and GIV expression combined serve as a better prognosis marker in NSCLC patients than either marker alone.

## Discussion

Tumor angiogenesis, the formation of new capillaries from the existing vascular network, is essential for tumor growth and metastasis. However, the precise molecular events that initiate this complex process of angiogenesis in NSCLCs are poorly understood. In the tumor microenvironment, inflammatory cells and molecules influence almost every aspect of cancer progression. We and others have previously demonstrated that suggests IL-17 is elevated in several types of cancer, but how IL-17 might contribute to tumor angiogenesis is still unclear. Here, we found that IL-17 selectively augments the secretion of various angiogenic factors in tumor cells and promotes endothelial cell tube formation. We suggest that IL-17 mediates these effects by activating STAT3/GIV signaling and by subsequently up-regulating the STAT3/GIV downstream target VEGF. Consistent with these findings, in human NSCLC tissues, IL-17 expression was significantly and positively associated with GIV and increased tumor vascularity. Our findings thus support the notion that IL-17 can promote NSCLC angiogenesis.

Previous studies have shown that IL-17 has diverse effects on inflammatory cells and stromal cells, with most effects relating to angiogenesis stimulation and inflammation^13^,^14^,^30^. In our study, HUVECs treated with IL-17 showed increased formation of vessel-like tubes, and IL-17 expression significantly correlated with MVD in human NSCLC tissues. Recently, IL-17 has also been reported to mediate the release of proinflammatory factors and chemokines from tumor cells, including renal, hepatocellular and colorectal cancers^20^,^29^,^31^. Here, IL-17R was highly expressed in all NSCLC cell lines. We thus hypothesize that IL-17-mediated promotion of tumor angiogenesis involves an effect on NSCLC cells, which up-regulate their production of proangiogenic factors. In support of this hypothesis, we found that exogenous IL-17 stimulation increases IL-6 and VEGF levels in all NSCLC cell lines studied, increased IL-8 expression in H460 and H520 cells and decreased expression of Endostain in A549 cells.

In numerous inflammatory cells and tumor cells, IL-17 mediates signaling through distinct pathways, such as the MAPK, NF-κB, and STAT3 pathways^32^. Previously, IL-17 has been reported to stimulate production of IL-6 and STAT3 activation in inflammatory cells and fibroblasts in an autoimmune disease^33^ as well as in cancer cells^24^,^34^. Furthermore, the STAT3 signaling pathway controls a number of important biological responses, including immune functions, cellular growth, cellular differentiation and hematopoiesis^24^. In this study, we found that IL-17 could stimulate the production of IL-6 and STAT3 activation in NSCLC cells. Moreover, we observed that in HUVECs incubated with CCM from A549-siRNA-STAT3 cells, the ability of IL-17 to promote tube formation was diminished, as evidenced by reduced tube length. This result suggests an important contribution from STAT3 in IL-17-mediated tumor angiogenesis. In a wide range of cancers, immunohistochemical studies have indicated that VEGF expression is correlated with elevated STAT3 activity^15^,^35^,^36^. Indeed, STAT3 has been shown to be a direct transcriptional activator of the VEGF gene^37^. Of the various factors involved in angiogenesis, members of the VEGF family have predominant roles^6^,^38^. In our present study, HUVEC tube formation assays show that CCM from A549 cells treated with IL-17 significantly enhanced tube formation, while VEGF mAbs significantly reversed the IL-17-stimulated tube formation. However, different cell types appear to respond differently to IL-17 in terms of target gene expression. Although IL-8 has been reported to be affected by IL-17 in other cell types^29^,^39^, cytokines in all NSCLC cells were not significantly altered in the presence of IL-17.

STAT3 is a downstream target of several cell surface receptors that can be activated by a plethora of soluble mediators, including interleukins (IL-3, IL-6, IL-10) as well as other cytokines(G-CSF, EGF, FGF, PDGF) and hormones (growth hormone, leptin)^28^,^29^,^40^,^41^. We found that IL-17 up-regulates IL-6 expression in NSCLC cell lines. When we used neutralizing IL-6 treated with IL-17 induced cells, we found that IL-17-induced STAT3 activation and increased the tube length were diminished. By contrast, the production of IL10, bFGF and PDGF were not significantly affected by exogenous IL-17. Our study showed that IL-6 may be a main upstream activator of pSTAT3 expression, but is not indispensable in the setting of IL-17 stimulation. However, it is true that pSTAT3 expression still increases in response to IL-17 after neutralizing IL-6 (figure S7). As an explanation for this result, this increase could be due to other known STAT3 activators that could be produced by IL-17 treatment of tumor cells. More recently, Hu *et al.* demonstrated that TGF-β increased the levels of STAT3 in prostate cancer cells^42^ and another reports suggested that STAT3 was activated by G-CSF in myeloma and ovarian cancer cells^43^,^44^. Importantly, although GSM-CSF, G-CSF, TNF-α, TGF-β have been reported to be affected by IL-17 on other cell types^45^, production of these cytokines in NSCLC cells remain elusive and should be investigate in future studies.

Recently, Dunkel and colleagues have shown that GIV is a direct target of STAT3 and that GIV positively autoregulates its own transcription by enhancing STAT3 activation via its GEF motif^17^. Our results show that IL-17 induces angiogenesis in NSCLC cells by activating the STAT3/GIV signaling pathway and that knockdown of STAT3 significantly reduces GIV expression. Furthermore, others also found GIV to be an important metastasis-related protein in the progression of diverse cancers^26^,^46^,^47^. In our study, GIV expression in A549 cells is important for IL-17-induced endothelial cell tube formation, and GIV expression is positively correlated with IL-17 and MVD in human NSCLC tissue. These results provide a mechanistic explanation for the prognostic studies that have directly linked IL-17, STAT3 signaling, and GIV with tumor recurrence, tumor metastasis, and poor survival in cancer patients. To the best of our knowledge, this study is the first to demonstrate that IL-17-induced STAT3/GIV activation occurs during tumor cell angiogenesis.

Previous results have shown a protumor effect of IL-17 in hepatocellular and colorectal carcinoma; IL-17 was shown to be an independent prognostic factor for OS and DFS^21^,^29^. Consistent with these previous results, we found that IL-17 is associated with poor prognosis and that intratumoral IL-17-producing cells are positively and significantly correlated with MVD in human NSCLC tissues. Although consistent with several recent publications regarding the role of IL-17 in promoting tumor progress, these findings contradict other reports suggesting that IL-17 can provide an antitumor effect against certain tumors^8^,^30^. Furthermore, GIV expression positively correlated with the presence of IL-17+ cells, and both were associated with the presence of microvascular invasion and poor survival in NSCLC patients. The combination of intratumoral IL-17+ cells and GIV expression served as a better predictor of poor survival than either alone in NSCLC patients.

In conclusion, our results suggest that IL-17-mediated tumor angiogenesis involves activation of the STAT3/GIV signaling pathway and subsequent up-regulation of VEGF production in NSCLC cells. These might be the mechanisms that underlie the correlation between IL-17 and poor prognosis and angiogenesis. Therapies that target IL-17 and GIV may be developed as potential therapeutic approaches to inhibit NSCLC.

## Materials and Methods

### Patients

A total of 67 patients who underwent surgery for histologically verified NSCLC at the Department of Pathology, The Harbin Medical University Cancer Hospital between 2006 and 2010 were enrolled in this study. None of the patients received any anticancer therapy prior to sample collection. The tumor stage was determined according to the 2010 American Joint Committee on Cancer and International Union Against Cancer tumor-node-metastasis (TNM) classification system. Tumor differentiation was graded according to the Edmondson and Steiner grading system. All experiments were performed in accordance with the relevant guidelines and regulations of Harbin Medical University. And this study was approved by the Ethics Committee of Harbin Medical University, and written informed consent was obtained from each patient.

### Cell culture

The human lung adenocarcinoma cell lines A549, H1792 and H522, lung squamous carcinoma cell line H520 and H226, large cell lung cancer cell line H460, and HUVECs were purchased from American Type Cell Collection (ATCC, Manassas, VA, USA). Lung cancer cells were cultured in RPMI 1640 (Gibco, USA) supplemented with 10% fetal calf serum. HUVECs were maintained using the EGM-2 bullet kit (Lonza, Basel, Switzerland) in a humidified atmosphere of 5% CO_2_ at 37 °C.

The NSCLC cells were treated with medium alone or with various concentrations (10 ng/ml, 50 ng/ml, and 100 ng/ml) of recombinant human interleukin-17 (R&D Systems, Minneapolis, MN, USA) in RPMI-1640 medium for 24 h or at 100 ng/ml for the indicated time. To prevent the effects of cytokines, IL-17 mAb, IL-6 mAb and VEGF mAb (R&D Systems, Minneapolis, MN, USA), were added to the IL-17 treatment medium.

### Transient transfection with siRNA

STAT3- and GIV- siRNAs, as well as negative control RNA molecules with mismatched sequences, were synthesized by Oligofectamine (Invitrogen,San Diego, CA) using the following sense and anti-sense strands: STAT3 sense, 5′ GGGACCUGGUGUGAAUUAUTT 3′ and antisense, 5′ AUAAUUCACACCAGGUCCCTT 3′; GIV sense, 5′ GAGGCAGACAGUGUCAUUATT 3′; antisense, 5′ UAAUGACACUGUCUGCCUCTT 3′. Transfection into NSCLC cells was performed using Lipofectamine™ 2000 (Invitrogen) according to the manufacturer’s instructions.

### Western blot analysis

The cells were lysed with lysis buffer (Cell Signaling Technology, Danvers, MA, USA) containing a protease inhibitor (Sigma Chemical company, St. Louis, MO, USA). Protein concentration was quantified using the BCA protein assay kit (Santa Cruz, USA). Western blotting was performed as previously described^48^. Specific primary antibodies against p-STAT3 (Y705), STAT3 (Cell Signaling Technology, Beverly, MA), and GIV (Millipore, MA, USA) were used in our study. Tubulin (Sigma, USA) was used as a loading control.

### Real-time RT-PCR analysis

Total RNA from the cultured lung cancer cells was extracted using the Trizol reagent (Invitrogen, Carlsbad, CA, USA) according to the manufacturer’s protocol. Complementary DNA was synthesized from 1 ng of total RNA using the PrimeScriptTM RT reagent kit with gDNA Eraser (Takara Bio, Inc, Dalian, China) in a final reaction volume of 20 μl. Semiquantitative real-time PCR was performed using the SYBR Green Master Mix (Roche Applied Science, Mannheim, Germany) on an ABI7500 Sequence Detection System (Applied Biosystems, Foster City, CA, USA). Glyceraldehyde 3-phosphate dehydrogenase (GAPDH) was used as the internal control to correct for variations in the cDNA content among the samples. The primers were synthesized by Oligofectamine (Invitrogen, San Diego, CA). No amplification of nonspecific products was observed in any of the reactions, as determined from an analysis of the dissociation curves. The data were normalized to the GAPDH expression levels and are presented as the averages from three repeated experiments. The relative gene expression levels were calculated using the comparative Ct (△△Ct) method; the relative expression is calculated as 2^−△△Ct^, where Ct represents the threshold cycle.

### Conditioned medium and ELISA

Supernatants from NSCLC cells treated with or without IL-17 (100 ng/ml) for 24 h were collected. The medium was centrifuged to remove the cellular debris and frozen at −80 °C until subjected to ELISA to assay levels of angiogenic factor. Angiogenic factor concentrations were determined using a commercial ELISA kit (Uscn Life Science, Houston, USA) according to the manufacturer’s instructions. The level of angiogenic factor was expressed in picograms per milliliter.

### Immunohistochemistry and assessment

Immunohistochemistry to detect IL-17, CD34 and GIV expression was performed as previously described^49^. The primary antibodies and dilutions were as follows: rabbit polyclonal anti-IL-17 antibody (1:100, Santa Cruz Biotechnology, Santa Cruz, CA, USA), rabbit polyclonal anti-GIV antibody (1:100, Millipore, MA, USA) and rabbit monoclonal anti-CD34 antibody (1:200, Zhongshan Company, Beijing, China). The negative control sections were treated with PBS instead of the primary antibodies.

Stained tissue sections were evaluated using light microscopy at 200× or 400× magnification by two pathologists. Five representative fields of each case were captured. To calculate the percentages of IL-17- and GIV-positive cells, the number of positive-staining cells and total number of cells in five count areas of each photograph were measured using Image-Pro Plus v6.0 software (Media Cybernetics, Inc.). The mean percentage of positive cells was calculated as the quotient of the number of positive cells divided by the total number of cells. The MVD was assessed via immunohistochemistry using a CD34 marker. The stained sections were screened at 200× magnification for hot spots, and the average number of microvessels was recorded. Any red-staining cells that were morphologically compatible with endothelial cells and that were in a cluster containing or lacking a lumen (rudimentary or well-formed) were considered microvessel cells and were counted as a vessel. Two observers were responsible for the microvessel number counting, and the mean value was used for analysis.

### Immunofluorescence staining

A549 cells and H520 cells were cultured in slides within 6-well plates. Tumor cells and siRNA-treated tumor cells were treated with recombinant IL-17 (100 ng/mL) for 24 h. At the end of the incubation, cells were fixed with 4% paraformaldehyde for 15 minutes at RT and permeabilized by treating with 100% methanol for 10 minutes at 20 °C. Next, slides were washed with PBS, blocked with normal goat IgG for 1 hour at room temperature and incubated overnight at 4 °C with rabbit anti-p-STAT3 or rabbit anti-GIV antibodies. After washing with PBS, chamber slides were incubated with secondary antibodies (goat anti-rabbit-IgG-Alexa Fluor 594). Slides were then mounted with Prolong Gold Antifade Reagent with 40,6-diamidino-2-phenylindole (DAPI; Invitrogen). The fluorescent images were analyzed by fluorescence microscopy.

### Tube formation assay

The tube formation assay was performed as described previously^50^. Briefly, a 96-well plate was coated with 60 μl of matrigel (BD Biosciences, USA), which was allowed to polymerize and solidify at 37 °C for 30 min. The HUVECs were seeded onto the matrigel layer in the presence or absence of various concentrations of IL-17 (10, 50, and 100 ng/ml) or conditioned cell media (CCM). After 8–16 h, blood-vessel-like tubules from three randomly chosen fields were counted and photographed under a microscope (Nikon, Japan). The tube length was quantified using the Image-Pro Plus v6.0 software (Media Cybernetics, Inc.). Results are represented as total tube length (μm) for three photographic fields per experimental condition. Each treatment was performed in duplicate and the experiment was independently repeated three times.

### Statistics

Statistical analysis was performed with SPSS 17.0 software (SPSS, Chicago, IL). Each treatment was performed in duplicate and the experiment was independently repeated three times. Measurement values were expressed as the means ± standard deviations. Student’s *t* test and Spearman’s *r* correlation were used as appropriate. The survival rates were performed by the Kaplan-Meier method (log-rank test). Cox multivariate analysis with a stepwise method (forward, likelihood ratio) was used to determine the independent prognostic factors. A *p* value < 0.05 was judged to indicate significant results. The median values of the percentage of IL-17+ cells and of GIV+ cells were used as cutoffs to dichotomize the immunostaining.

## Additional Information

**How to cite this article**: Pan, B. *et al.* Interleukin-17 promotes angiogenesis by stimulating VEGF production of cancer cells via the STAT3/GIV signaling pathway in non-small-cell lung cancer. *Sci. Rep.* **5**, 16053; doi: 10.1038/srep16053 (2015).

## Supplementary Material

Supplementary Information

## Figures and Tables

**Table 1:** IL-17 affects the production of proangiogenic factors of NSCLC.

Angiogenic factors	A549(pg/ml)			H520(pg/ml)		
	PBS	IL-17	p-value	PBS	IL-17	p-value
IL-6	21.0 ± 0.5	82.7 ± 5.2	<0.001	25.9 ± 0.7	66.0 ± 1.4	0.005
IL-10	35.2 ± 0.2	36.5 ± 0.3	0.106	44.7 ± 0.7	44.6 ± 0.6	0.239
IL-8	1052.5 ± 15.1	1048.4 ± 27.9	0.102	2706.1 ± 500.5	3436.1 ± 297.2	0.016
bFGF	42.9 ± 3.1	44.6 ± 3.4	0.569	50.6 ± 0.4	76.8 ± 0.6	0.172
VEGF	426.8 ± 35.3	2326.9 ± 103.0	<0.001	551.6 ± 13.4	2850.0 ± 40.9	0.016
PDGF	14.7 ± 0.5	16.1 ± 0.6	0.799	22.46 ± 0.6	22.0 ± 0.4	0.060
Endostain	25.4 ± 1.8	14.9 ± 1.3	0.047	38.0 ± 2.9	23.5 ± 0.5	0.139

ELISA-determined cytokine levels were exprssed as means ± SD. *p*-values were calculated by student’s *t* test.

**Table 2:** Univariate and multivariate analyses of factors associated with survival.

Variables	Univariate: *p*	Multivariate	
		HR (95% CI)	p
Gender(female vs. male)	NS	NA	NA
Age, years(<65 vs. ≥65)	NS	NA	NA
Smoking status(smoker vs. nonsmoker)	NS	NA	NA
TNM stage (I-II vs. III-IV)	<0.001	3.406(1.762–6.587)	<0.001
Differentiation (well vs. Moderate-poor)	NS	NA	NA
Histological type (ADC vs. non-ADC)	NS	NA	NA
IL-17 expression (low vs. high)	0.011	NA	NS
GIV expression (low vs. high)	0.030	NA	NS
Combination of IL-17 and GIV*			
Overall	0.003	NA	0.004
I vs II	0.011	3.141(1.484–6.647)	0.002
I vs III	0.005	2.544(1.154–5.606)	0.017

Note: Univariate analysis was calculated using the Kaplan-Meier method (the log-rank test). Multivariate analysis was performed using the Cox multivariate proportional hazard regression model with a stepwise method (forward, likelihood ratio).

Abbreviations: HR, hazard ratio; CI, confidence interval; ADC, adenocarcinom; NA, not assessed; NS, not significant; TNM, tumor-node-metastasis.

*I, IL-17 high and GIV high expression; II, IL-17 high, GIV low expression or IL-17 low, GIV high expression; III, IL-17 low and GIV low expression.
