# Author Correction: Interleukin-17 promotes angiogenesis by stimulating VEGF production of cancer cells via the STAT3/GIV signaling pathway in non-small-cell lung cancer

**DOI:** 10.1038/s41598-020-65650-5

**Published:** 2020-05-27

**Authors:** Bo Pan, Jing Shen, Jingyan Cao, Yongxu Zhou, Lihua Shang, Shi Jin, Shoubo Cao, Dehai Che, Fang Liu, Yan Yu

**Affiliations:** 1 Department of Medical Oncology, Harbin Medical University Cancer Hospital, Harbin, 150081 P.R. China; 2 Department of general surgery, The Fourth Affiliated Hospital of Harbin Medical University, Harbin, 150001 P.R. China

Correction to: *Scientific Reports* 10.1038/srep16053, published online 03 November 2015

This Article contains errors.

An incorrect blot for the time-dependent measure of p-STAT3 in A549 cells was used in Figure 2b. An incorrect blot for STAT3 (A549 and H1792 cells) was used in Figure 3a. An incorrect blot was used for the GIV blot (A549 cells) in Figure 3c. The correct versions of these panels appear below as Figure 1.

**Figure 1:**
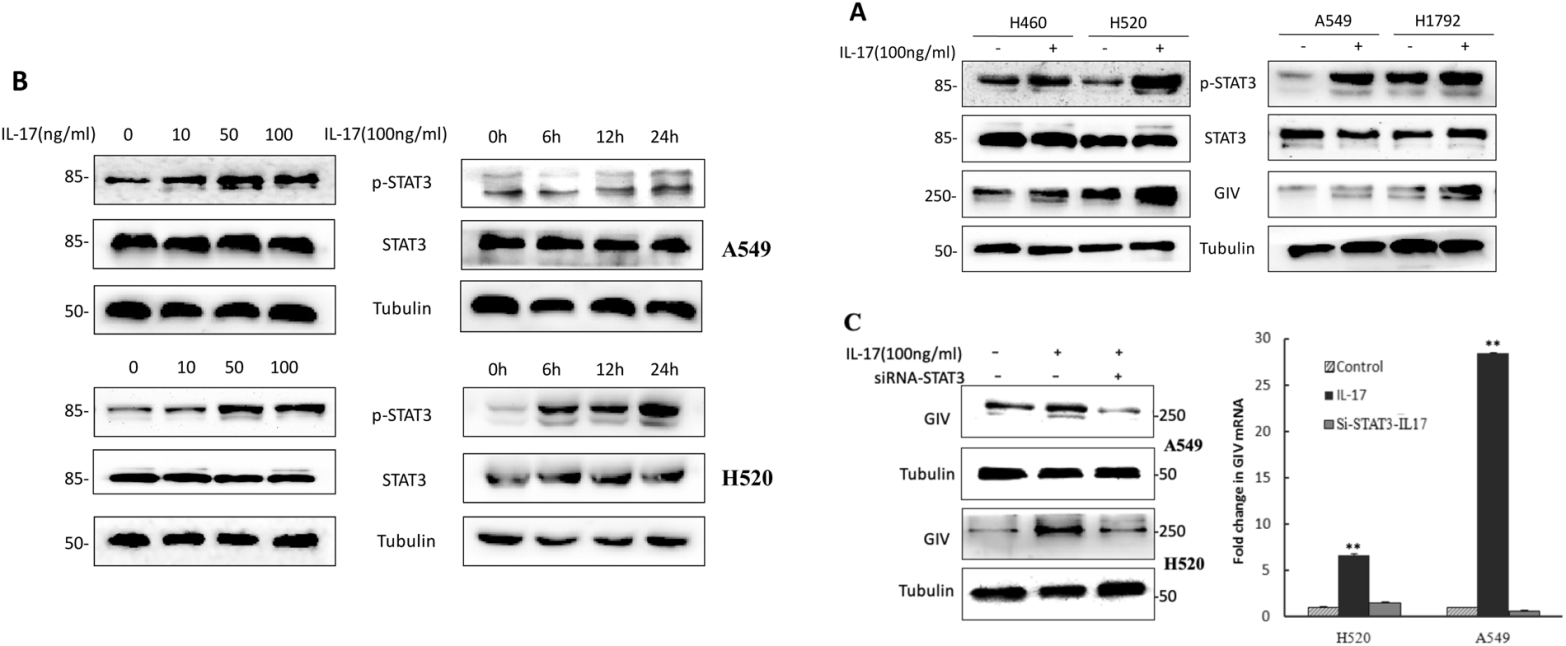
.

The Supplementary Information did not include full-length gel images. Uncropped images for the blots used in Figures 2b, 3a, and 3c, are provided below as Figure 2.

**Figure 2:**
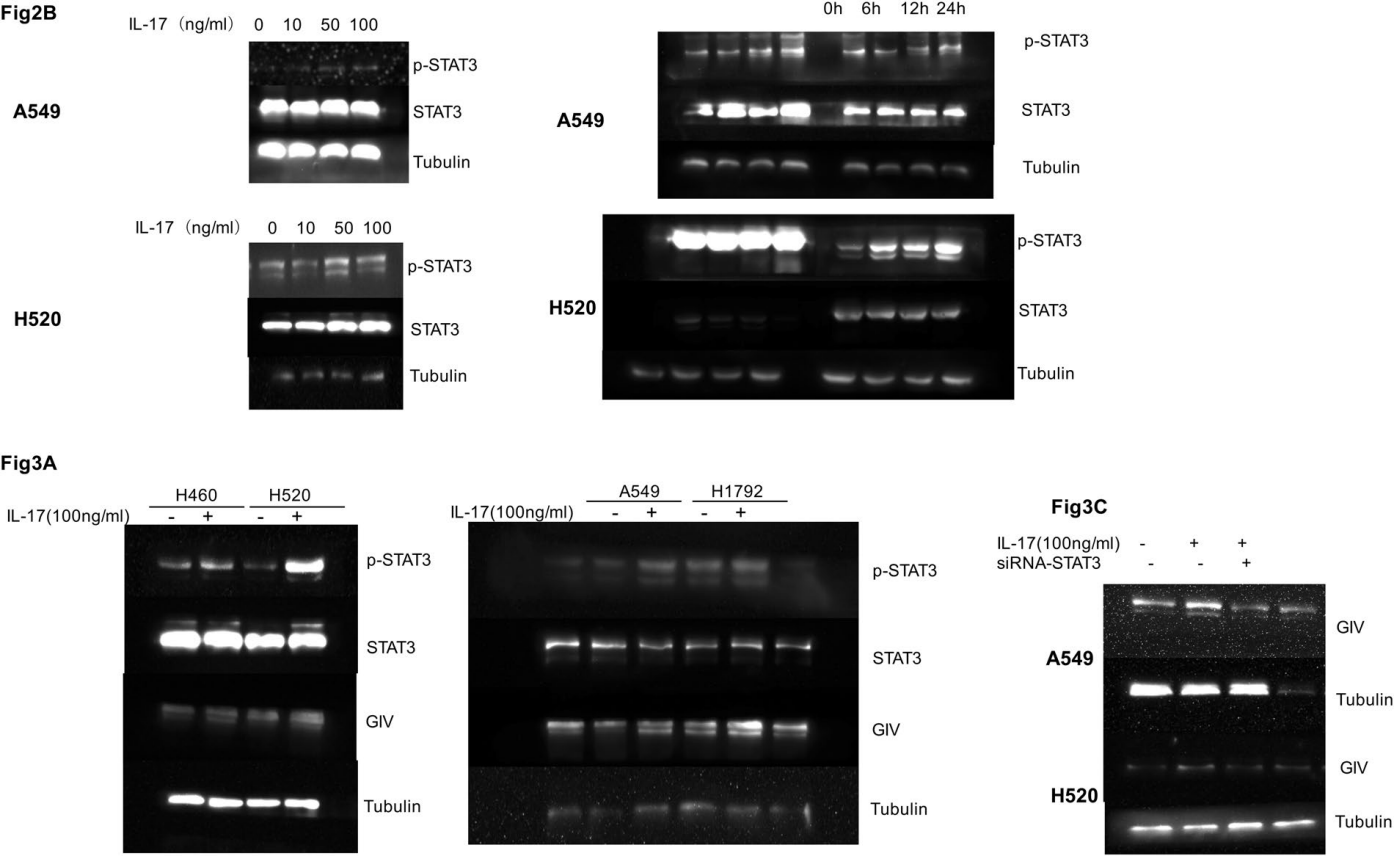
.

