# Supplementary material for: Interleukin-17 promotes angiogenesis by stimulating VEGF production of cancer cells via the STAT3/GIV signaling pathway in non-small-cell lung cancer
Source: Sci Rep. 2015 Nov 3;5:16053. doi: 10.1038/srep16053 (PMC4630609; doi:10.1038/srep16053)
Supplement: Supplementary Information [file srep16053-s1.pdf]

Interleukin-17 promotes angiogenesis by stimulating VEGF production of cancer cells via the STAT3/GIV signaling pathway in non-small-cell lung cancer

Pan B[1], Shen J[1], Cao JY[1], Zhou YX[2], Shang LH[1], Jin S[1], Cao SB[1], Che DH[1], Liu F[1], Yan Y[1]

[1]Department of Medical Oncology, Harbin Medical University Cancer Hospital, Harbin 150081, PR China

[2]Department of general surgery, The Fourth Affiliated Hospital of Harbin Medical University, Harbin 150001, PR China

Correspondence and requests for materials should be addressed to Y. Yu (email: yuyan1291@126.com)

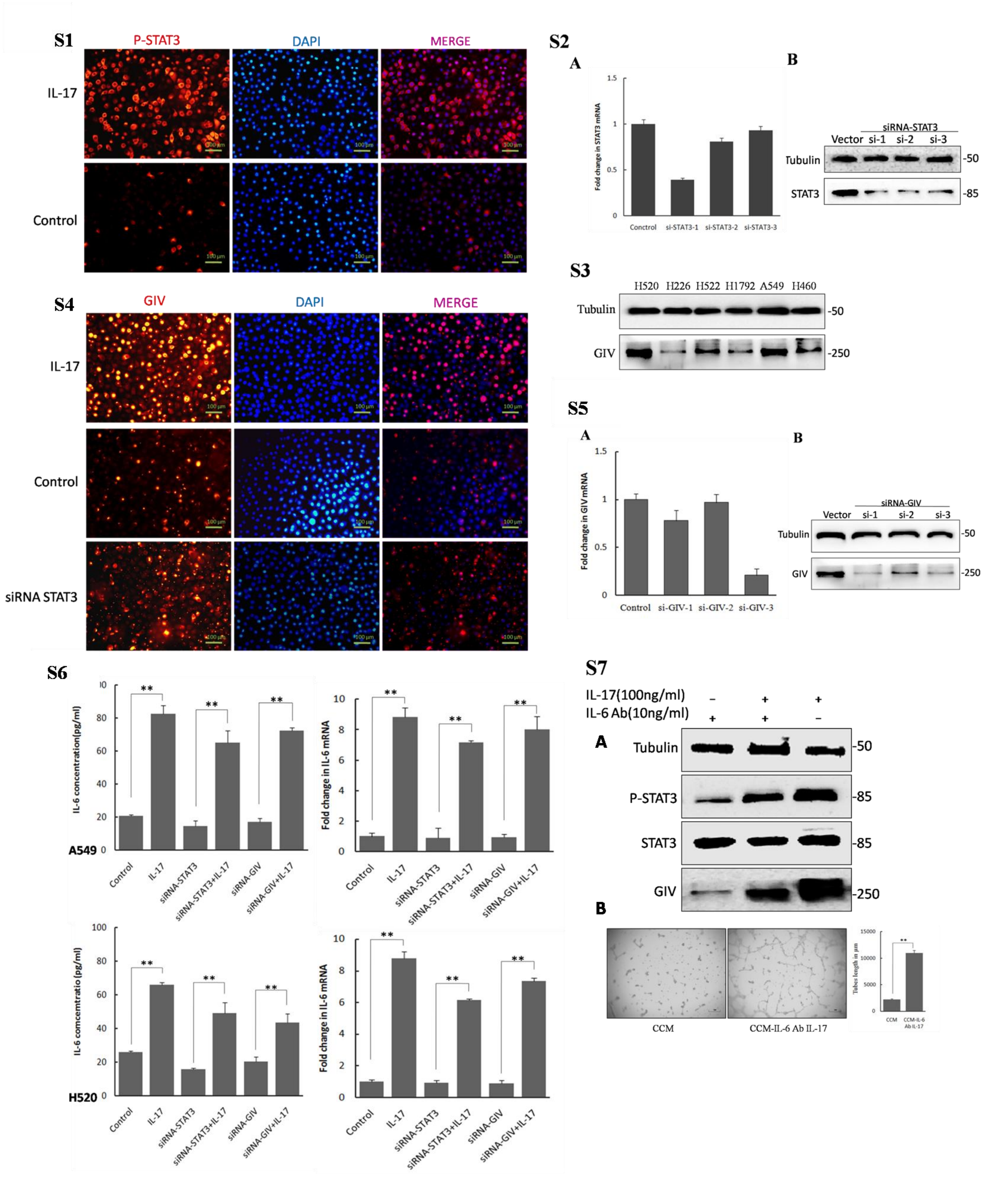

Figures legends

Fig.S1 Immunofluorescence assays showed that recombinant human IL-17(100ng/ml for 24h) significantly elevated the expression of p-STAT3 in H520 cells. Photomicrographs were taken at ×200 magnification. Control, PBS.

Fig.S2 A549 cells were transfected with control or STAT3 siRNAs. The cells were incubated for 48 h, and the cell lysates were analyzed by (A) real-time PCR and (B) Western blot analyses.

Fig.S3 Western blotting showed that expression of GIV in NSCLC cell lines.

Fig.S4 Immunofluorescence assays showed that recombinant human IL-17(100ng/ml for 24h) significantly elevated the expression of GIV in H520 cells. However, the expression of GIV decreased if depleted of endogenous STAT3. The photomicrographs were taken at ×200 magnification.

Fig.S5 A549 cells were transfected with control or GIV siRNAs. The cells were incubated for 48 h, and the cell lysates were analyzed by (A) real-time PCR and (B) Western blot analyses.

Fig.S6 NSCLC cells were cultured for 24h with IL-17(100ng/ml) or PBS. Concentrations of IL-6 in culture supernatants were measured by ELISA, and mRNA of IL-6 in NSCLC cells were measured by Real-time PCR. IL-17 up-regulated the production of IL-6 by tumor cells, while both in siRNA-STAT3 and siRNA-GIV cells, IL-17-induce d IL-6 upregulation was not affected.

Fig.S7 IL-17 partially activates STAT3 and increased tube formation via IL-6 induction. A549 cells were treated with control, IL-17 or IL-17 and IL-6 mAb (5ng/ml). Cell lysates were analyzed by western blot(A) and CCM were harvested added to HUVECs plated on Matrigel for tube formation assay(B).

Table S1 IL-17 affects the production of proangiogenic factors of NSCLC

	H1792(pg/ml)			H460(pg/ml)		
Angiogenic factors	Control	IL-17	p-value	Control	IL-17	p-value
IL-6	47.7±4.5	83.4±10.2	0.040	43.9±0.4	103.0±1.8	＜0.001
IL-10	28.8±0.7	28.9±0.8	0.421	23.8±2.0	24.2±1.4	0.102
IL-8	3353.5±14.0	2789.1±22.8	0.126	110.3±10.2	388.9±30.9	0.050
bFGF	65.2±2.6	84.3±0.5	0.100	87.8±2.3	84.3±3.7	0.054
VEGF	570.5±17.6	1069.9±5.7	0.012	493.9±1.1	1772.1±19.5	0.043
PDGF	15.5±0.4	12.3±0.3	0.293	15.0±1.6	20.6±2.0	0.658
Endostain	41.8±0.1	31.3±0.3	0.114	36.4±0.1	21.5±0.1	0.463

ELISA-determined cytokine levels were exprssed as means±SD. *p*-values were calculated by student's *t* test.

Table S2 IL-17 and GIV expression in NSCLC and the relationship with patients' characteristics.

		IL-17			GIV		
Pathological features	Total	Low	High	*p*-value	Low	High	*p*-value
Age(years)							
＜65	36	19	17		21	15	
≥65	31	12	19	0.250 [a]	12	19	0.109 [a]
Gender							
Female	27	12	15		12	15	
Male	40	19	21	0.806 [a]	21	19	0.518 [a]
Smoking status							
Smoker	49	23	26		24	25	
Non-smoker	18	8	10	0.856 [a]	9	9	0.941 [a]
Tumour stage							
I and II	35	21	14		19	16	
III and IV	32	10	22	0.018 [a]	14	18	0.389 [a]
Histological type							
Squamous cell carcinoma	26	10	16		12	14	

Adenocarcinoma	33	17	16		18	15	
Others	8	4	4	0.580[b]	3	5	0.657[b]
Differentiation							
Well differentiated	24	12	12		10	14	
Moderately-poorly	43	19	24	0.647[a]	23	20	0.353[a]

[a] Pearson's χ^2 test.

[b] Fisher's exact test

Table S3 Univariate and multivariate analyses of factors associated with survival

Variables	A		B		C	
	HR (95% CI)	*p*	HR (95% CI)	*p*	HR (95% CI)	*p*
Gender(female vs. male)	NA	NA	NA	NA	NA	NA
Age, years(＜65 vs. ≥65)	NA	NA	NA	NA	NA	NA
Smoking status(smoker vs. nonsmoker)	NA	NA	NA	NA	NA	NA
TNM stage (I-II vs. III-IV)	0.311(0.165-0.586)	＜0.001	0.293(0.154-0.557)	＜0.001	0.294(0.152-0.568)	＜0.001
Differentiation (well vs. Moderate-poor)	NA	NA	NA	NA	NA	NA
Histological type (ADC vs. non-ADC)	NA	NA	NA	NA	NA	NA
IL-17 expression (low vs. high)	NA	NS	NA	NA	NA	NA
GIV expression (low vs. high)	NA	NA	0.467(0.250-0.872)	0.016	NA	NA
Combination of IL-17 and GIV*						
Overall	NA	NA	NA	NA	NA	0.004
I vs II	NA	NA	NA	NA	3.141(1.484-6.647)	0.002
I vs III	NA	NA	NA	NA	2.544(1.154-5.606)	0.017

Note: Multivariate analysis was performed using the Cox multivariate proportional hazard regression model with a stepwise method (forward, likelihood ratio).

Abbreviations: HR, hazard ratio; CI, confidence interval; ADC, adenocarcinom; NA, not assessed; NS, not significant; TNM, tumor-node-metastasis.

*I, IL-17 high and GIV high expression; II, IL-17 high, GIV low expression or IL-17 low, GIV high expression; III, IL-17 low and GIV low expression.
